# Neural Correlates of Cognitive Disengagement Syndrome Symptoms in Children: A Magnetoencephalography Study

**DOI:** 10.3390/brainsci15060624

**Published:** 2025-06-10

**Authors:** Xiaoqian Yu, Jing Xiang, Jeffery N. Epstein, Leanne Tamm, Josalyn A. Foster, Stephen P. Becker

**Affiliations:** 1 School of Psychology, Wenzhou-Kean University, Wenzhou 325060, China; xiaoqyu@kean.edu; 2 Department of Pediatrics, University of Cincinnati College of Medicine, Cincinnati, OH 45221, USA; jing.xiang@cchmc.org (J.X.); jeff.epstein@cchmc.org (J.N.E.); leanne.tamm@cchmc.org (L.T.); 3 Division of Neurology, Cincinnati Children’s Hospital Medical Center, Cincinnati, OH 45229, USA; 4 Division of Behavioral Medicine and Clinical Psychology, Cincinnati Children’s Hospital Medical Center, Cincinnati, OH 45229, USA; 5 School of Human Services, University of Cincinnati, Cincinnati, OH 45221, USA; fostej8@mail.uc.edu

**Keywords:** cognitive disengagement syndrome, sluggish cognitive tempo, ADHD, MEG, attention network test

## Abstract

**Background/Objectives:** Despite the growing recognition of cognitive disengagement syndrome (CDS), previously termed sluggish cognitive tempo, as a distinct dimension of psychopathology, the neural correlates of CDS remain largely unknown. We investigated the neural correlates of CDS in children using whole-head magnetoencephalography (MEG). **Methods**: A community-based sample of children (*N* = 43, ages 8–12 years) was recruited and completed self-report ratings of CDS. MEG was recorded while the children completed an adapted version of the attention network test (ANT). **Results**: The results indicated that higher levels of self-reported CDS symptoms were associated with larger changes in the root-mean square (ΔRMS) (incongruent—congruent trials) in M2 and M3, suggesting children with higher levels of CDS symptoms might require greater mental effort to overcome distractors during incongruent trials. The source localization analysis initially revealed a negative correlation between child self-reported CDS symptoms and ΔM2 power (incongruent—congruent trials) in the medial prefrontal cortex (mPFC), suggesting insufficient power allocation in a region critical for attentional processing. However, this association was no longer significant after controlling for ADHD status. No significant correlation was found between self-reported CDS symptoms and alerting or orienting. **Conclusions**: These findings provide initial evidence of the disrupted attentional processing associated with CDS in children. Further replication and extension with larger samples are warranted.

## 1. Introduction

Cognitive disengagement syndrome (CDS), formerly termed sluggish cognitive tempo, describes a set of behaviors marked by excessive daydreaming, mind wandering, slowness in thinking, and mental confusion/fogginess [1]. Previous research has shown that these behaviors are distinct from, though related to, attention-deficit/hyperactivity disorder (ADHD) symptoms and other psychopathology dimensions, warranting more research [2]. Furthermore, children with CDS often experience a range of difficulties in their academic performance, social interactions, and overall well-being [1,3,4].

A small and methodologically varied literature has examined CDS in relation to neurocognitive performance, including processing speed [5,6,7], cognitive control [8], and executive function [9,10]; see review [11]. Given the historical links to ADHD inattention and symptom presentation involving excessive daydreaming [12], the link between CDS and neuropsychologically assessed attention has been of particular interest. A meta-analysis with 73 studies found that CDS was related to lower levels of sustained attention as assessed in several neuropsychological tasks [2]. Baytunca et al. (2018) also suggested that a sample of children with ADHD and CDS had greater difficulty in sustained attention than children diagnosed with ADHD alone [13].

While these findings point to important neurocognitive correlates of CDS, they also underscore the need to better understand the underlying neural mechanisms that give rise to these attentional difficulties. Despite growing research interest in CDS, the neural correlates of CDS remain largely unknown. The largest and most comprehensive neuroimaging study on CDS to date, conducted among 178 Spanish schoolchildren, found a combination of gray and white matter enlargements located in or near the frontal eye fields, which are part of the dorsal attention network, as well as in the right and left frontal operculum [14]. Additionally, a study using a cued flanker task in a sample of ten adolescents found that CDS symptoms were associated with hypoactivity in the left superior parietal lobe (SPL) in response to cues, indicating potential impairment in reorienting or shifting attention [15]. A recent study compared the neurovascular bases of ADHD and CDS, finding a weak negative correlation between right internal carotid (ICA) blood flow volume (BFV) and CDS scores. Patients with CDS also showed lower right and total ICA BFV than those in the ADHD and control groups and a weak negative correlation between right ICA BFV and CDS scores. These results suggest that CDS is a unique phenotype of attention difficulties with distinct neurobiological characteristics from ADHD [16].

Research on the neural correlates of CDS should be grounded in theoretical frameworks that are relevant to its underlying processes, such as Posner’s model of attention. This well-established framework proposes that the attentional network consists of three key components: the alerting, orienting, and executive control networks [17]. The alerting network helps maintain a state of vigilance and increases physical readiness to respond to external warning signals; the orienting network facilitates disengaging attention from the current focus and shifting it to a new target; the executive control network enables the inhibition of automatic responses and resolves conflicts between competing mental processes, allowing for control over thoughts or behaviors. All three attention networks incorporate both bottom-up and top-down mechanisms, though the degree and the nature of this involvement vary by network and task context [17,18]. For instance, alerting can involve bottom-up processes such as phasic arousal in response to external cues, as well as top-down regulation in tonic alertness to maintain readiness over time [17,19]. Similarly, orienting may be driven by bottom-up exogenous cues (e.g., flash light) or top-down endogenous cues (e.g., symbolic direction), depending on the task demands [20]. Importantly, lower alerting or orienting scores may reflect enhanced top-down control, suggesting reduced reliance on external cues and more self-regulated attentional allocation [19]. Additionally, the orienting score can involve both the dorsal attention network (associated with voluntary, goal-directed orienting) and the ventral attention network (linked to stimulus-driven shifts), depending on how the orienting index is derived [20]. In contrast, executive control primarily reflects top-down processes, though salient distractors can also engage bottom-up influences that increase conflict-monitoring demands [17,21].

Despite multiple studies employing the attention network test (ANT)—a task designed to assess the three networks within Posner’s model of attention—to examine attentional functioning in CDS, the results have been inconsistent across age groups and samples. Two studies in primary-school children from a general population found no significant association between CDS symptoms and any of the three attentional networks [22,23]. Another study in adults reported a weaker orienting network in the CDS groups than in the ADHD group and control group but found no difference in the alerting network among the groups [24]. To the contrary, one study found the presence and severity of CDS to be negatively associated with alerting but not with orienting or executive control in children and adolescents with ADHD (*n* = 107), who were either medication-naïve or washed-out prior to testing, and in typically developing controls (*n* = 30) [25].

To our knowledge, no neuroimaging study to date has investigated the neural correlates of self-reported attentional functioning in relation to CDS in children. This is important given the internal nature of CDS involving excessive daydreaming and mind-wandering [26]. We aimed to add to this area by examining CDS in relation to brain neuromagnetic activation using whole-head magnetoencephalography (MEG) while children were performing the ANT. MEG is a noninvasive neuroimaging technique used to measure magnetic fields produced by neuronal electrical activity in the brain, featuring excellent temporal and spatial resolution [27].

## 2. Materials and Methods

### 2.1. Participants

This is a cross-sectional study. The sample was based on convenience sampling. The participants were 62 children (36 boys, 26 girls) aged 8–13 years (M = 9.89, SD = 1.43). The majority of the children were non-Hispanic White (*n* = 52), with the remaining participants being non-Hispanic Black (*n* = 8) or multiracial (*n* = 2). Incorrect and no-response trials, along with trials with a reaction time (RT) < 100 ms or >1040 ms, were removed (17%). Participants (*n* = 7) with an accuracy lower than 66% (mean—1 SD) were excluded from further analysis. Participants (*n* = 8) with <50% clean trials were also excluded from further analysis. Three participants were missing Digital Imaging and Communications in Medicine (DICOM) files necessary for MRI co-registration and localization, and one participant had technical difficulties during image acquisition, leaving forty-three participants (age, M = 9.95, SD = 1.45; 17 female) in the analyses. Based on the Kiddie Schedule for Affective Disorders and Schizophrenia (K-SADS) interview conducted with the child’s parent, 26 children met the criteria for ADHD (see Table 1 for ADHD presentation information). Eight of the children were taking a psychostimulant medication for ADHD, though all the participants had a 24 h washout before the scan. All the participants were right-handed based on the Edinburgh Handedness Inventory [28]. All the participants were required to meet the following inclusion criteria: be between ages 8 and 12 years; have an estimated verbal or composite IQ ≥ 80 on the Kaufman Brief Intelligence Scale, Second Edition [29]; possess sufficient English language skill to complete study measures; and, if applicable, be willing to discontinue any stimulant medication for 24 h before the study visits. The exclusion criteria were parent-report of a previous diagnosis of psychosis, bipolar disorder, obsessive–compulsive disorder, or autism spectrum disorder; taking any non-stimulant psychiatric medication and/or a history of epilepsy or head trauma associated with a loss of consciousness; or contraindications for scanning (e.g., metal, wearing braces).

### 2.2. Procedures

This study was approved by the Institutional Review Board (IRB) at Cincinnati Children’s Hospital Medical Center. Children were recruited for a CDS-focused study, with the full continuum of CDS symptom severity recruited via flyers distributed in the medical center, to schools, and pediatricians in the community and via social media advertising. Parents who contacted the research staff in response to the recruitment materials were given additional information and administered a phone screen to assess initial eligibility. At the inclusion/exclusion evaluation, all the parents signed informed consent, and the youth provided assent. The children completed the IQ screen, ANT, and CDS ratings, and the caregivers completed the KSADS interview. The eligible children were provided with a brochure that provided an overview of what to expect during the imaging session, and the parents were provided with an information sheet with similar information. In advance of the imaging appointment, the children viewed a short video that helped prepare them for the imaging visit and practiced lying still for 10 min periods, ideally under a chair or table. The imaging visit was typically scheduled within 1–2 months of the inclusion/exclusion evaluation.

### 2.3. Measures

**Child Concentration Inventory (CCI):** The Child Concentration Inventory (CCI) [30] was used to evaluate children’s self-reported CDS symptoms. The CCI comprises 14 items (e.g., “I get lost in my own thoughts”) rated on a four-point scale (0 = *not at all* to 3 = *very much*). As in previous research [31], 10 items from the CCI were used based on their established differentiation from ADHD inattention. A mean score was calculated (α = 0.77), with higher scores indicating greater CDS symptom severity.

**Figure 1:**
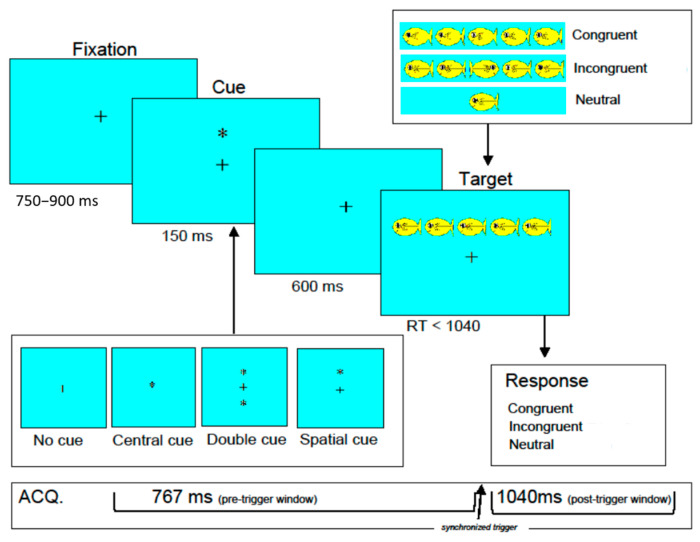
Structure of the child attention network test. +: a central fixation cross. *: asterisk in place of fixation cross (central cue trials), above and below fixation cross (double due trials), or in position of the upcoming stimulus (spatial cue trials).

**Attention Network Test (ANT):** The ANT combines a cued reaction time task [32] and a flanker task [33] to assess the efficiency of the three attention networks (i.e., altering, orienting, and executive) [18,19]. See Figure 1 for a diagram of the adapted version of the child ANT [34]. Each trial started with a central fixation cross of variable duration (between 750 and 900 ms), followed by a cue (150 ms), a fixation cross target stimulus (600 ms), and then the flanker stimuli (<1040 ms). There were four cue conditions that each appeared in 25% of trials: (1) no cue; (2) center cue (asterisk in place of fixation cross); (3) double cue (asterisks above and below fixation cross); or (4) spatial cue (single asterisk in position of the upcoming stimulus), 100% predictive of the target location. The flanker stimulus array was a set of one or five miniature fish presented horizontally that appeared either above the fixation (50%) or below the fixation (50%). There were three types of stimuli: congruent trials (33%; all five fish facing the same direction, either left or right), incongruent trials (33%; central fish facing the opposite direction from the flanking fish), and neutral trials (33%; one fish facing either left or right). Neutral trials were not examined in the current study. All the trials were randomly presented. The participants indicated the direction the central fish was facing by pressing the left or right button on the response box. After responding, they received auditory and visual feedback indicating accuracy. Feedback was provided 500 ms after the response, which consisted of an animation of the middle fish, showing it happy (blowing bubbles) and saying “yes” for correct responses or sad (tears coming down the eye) and saying “no” for incorrect or missed trials.

After orientation to the task, the children completed a 24-trial practice block and 1 experimental block of 96 trials at the inclusion/exclusion evaluation visit (out of the scanner). Following this practice, the children completed the ANT in the scanner at a subsequent imaging visit. To minimize head movement and tiredness during the scan, the 96 trials were randomly presented in a single block, and each participant completed four blocks. The participants were instructed to relax and rest for approximately one minute between the four blocks. Stimuli were back-projected onto a screen placed at the subjects’ feet and viewed via a mirror system mounted on the head-coil. The stimulus presentation and response collection were controlled using E-Prime Version 2.0 software (www.pstnet.com).

### 2.4. MEG Data Acquisition and Co-Registration with Structural MRI

MEG recordings were conducted using a 275-channel, whole-head MEG system (VSM MedTech Systems Inc., Coquitlam, BC, Canada) in a magnetically shielded room in the MEG Center at Cincinnati Children’s Hospital Medical Center. As in previous work [35], all the MEG data were recorded with third-order gradient noise cancellation. Prior to data acquisition, three small coils were attached to the participant’s nasion and left and right pre-auricular points. These three coils were subsequently activated at different frequencies to measure the participants’ head positions relative to the MEG sensors. The system allowed head localization to an accuracy of 1 millimeter (mm). The tolerable limit for head movement during MEG recording was 5 mm. The MEG recordings were sampled at 4 kHz, and each block (96 trials) was recorded as a single MEG dataset. Hardware filtering was turned off so that MEG data were recorded without online filtering.

Immediately following the MEG recording, three-dimensional Magnetization-Prepared Rapid-Acquisition Gradient Echo (MP_RAGE) sequences were obtained for all the participants with a 3T scanner (Siemens Medical Solutions, Malvern, PA, USA). Before the magnetic resonance imaging (MRI) scan, three fiduciary points were placed at positions identical to those of the three coils used in the MEG recordings to allow for an accurate co-registration of the MEG and MRI datasets.

MEG preprocessing and sensor-level analysis: The continuous magnetic time series was divided into epochs of 2000 ms duration (−500 to 1500 ms), with 0 ms defined as the flanker stimulus onset and the baseline defined as the −450 to −50 ms time window. Epochs containing artifacts were rejected based on a fixed-threshold method, supplemented with visual inspection. Artifact-free epochs were averaged separately over congruent and incongruent trials to generate grand-averaged waveforms. Consistent with the previous research [36,37,38], three event-related fields (ERFs) in the averaged waveform could be identified as magnetic responses: termed M1, M2, and M3. M1 was defined as the waveform during the interval between 50 and 150 ms after flanker stimulus onset. M2 was determined as the waveform during 150–250 ms after flanker stimulus onset. M3 was determined as the waveform during 250–350 ms after flanker stimulus onset. The root mean square (RMS), a statistical measure used to quantify the strength of the magnetic signals recorded from the brain, was obtained for the peak value in each ERF. Since M1 is less identifiable than M2 and M3 (*n* = 10, <25% of participants showed a clear M1), only the M2 and M3 amplitudes were analyzed. The signal power, i.e., the RMS magnitude across all MEG channels of the grand-average data, was calculated. Similar to obtaining attention network scores from RTs, ∆RMS was calculated by subtracting the RMS between conditions for each component, e.g., ∆RMS of M2 (incongruent—congruent) represents brain activation during conflict detection, and a higher value indicates more effortful conflict resolution since the brain needs to recruit more resources to handle conflict, possibly lowering efficiency in attentional processing. To identify the neuromagnetic biomarkers associated with CDS, a DC offset and filters were applied to all the data with the same settings for all the participants. As in previous studies [39,40], the RMS and latency of neuromagnetic responses were obtained with an MEG Processor with C/C+ on the Windows platform [41].

Source-level analysis: The MEG sensors were co-registered and adjusted to each participant’s structural MRI via the three fiducial points. A 3D brain mask was created with the MEG Processor to generate a realistic head model for the computation of the forward solution. The source locations corresponding to each response were estimated individually using beamforming. Details of the beamforming algorithms and steps are described elsewhere [35,42]. The combined imaging of the three-dimensional MEG sources and the MRI anatomical data was magnetic source imaging (MSI). The location and source strength (magnitude of source activation) were quantitatively measured for each MSI from each subject.

### 2.5. Data Analyses

Neutral trials were excluded from the analysis. Incorrect and no-response trials, along with trials with a reaction time (RT) < 100 ms or >1040 ms, were removed (17%). Participants (*n* = 7) with an accuracy lower than 66% (mean—1 SD) were excluded from further analysis. Participants (*n* = 8) with <50% clean trials were also excluded from further analysis [43]. Three participants were missing DICOM files necessary for MRI co-registration and localization, and one participant had technical difficulties during image acquisition, thus leaving forty-three participants (age, M = 9.95, SD = 1.45; 17 female) in the analyses. With these 43 participants, the ANT accuracy and median RT of correct trials were calculated for each subject since the RTs were not normally distributed. The score for each attention network was calculated according to previous research [19]: alerting (RT for no-cue trials minus RT for center-cue trials), orienting (RT for center-cue trials minus RT for spatial-cue trials), and executive (RT for incongruent trials minus RT for congruent trials).

All the statistical analyses were performed using the R 4.2.0 statistical package [44]. Descriptive statistics were obtained using the R package “psych” [45]. Separate linear mixed-effect models (LMEs) were conducted to examine reaction time and accuracy using the “lmer” function in the R package “lme4” [46], with cue and congruency entered as fixed effects and subjects as a random effect. Follow-up pairwise comparisons for LMEs were conducted using the “glht” function in R package [47] and corrected for multiple comparisons with the Tukey’s Honestly Significant Difference test. Spearman correlation analysis was conducted to examine the relation between CDS scores and ANT performance measured by attention network score and accuracy. Bonferroni correction was used to correct for multiple correlation comparisons. Of the 43 participants, 39 completed the CCI (M = 0.99, SD = 0.55). By controlling for ADHD status, partial correlations were used to examine the relationship between child self-reported CDS symptoms and behavioral outcomes of the ANT and measures of interest related to M2 and M3.

## 3. Results

### 3.1. Behavioral Data

#### 3.1.1. RT

Descriptive statistics of the median RT per ANT condition and their correlations with CDS symptoms are presented in Table 2. None of the RTs were significantly correlated with CDS symptoms. LME analyses revealed a main effect for cue; F(3, 120) = 79.24, *p* < 0.001, η^2^_p_ = 0.67. RTs in the no-cue condition were slower than RTs in other cue conditions (ps < 0.001 for spatial cue and double cue, *p* < 0.01 for center cue); RTs in the spatial-cue condition were faster than RTs in all other cue conditions (*p*s < 0.001). There was also a main effect for congruency; F(1, 40) = 147.48, *p* < 0.001, η^2^_p_ = 0.79. RTs in congruent trials (M = 638.74, SD = 13.03) were faster than RTs in incongruent trials (M = 685.98, SD = 13.45). There was a significant cue x congruency interaction effect; F(3, 120) = 3.45, *p* < 0.05, η^2^_p_ = 0.08. Specifically, RTs for congruent trials in the spatial-cue condition were faster than RTs in incongruent trials in the center-cue (*p* < 0.01), double-cue, and no-cue conditions (*p*s < 0.05). RTs in spatial-cue incongruent trials were slower than RTs in congruent trials under center-cue (*p* < 0.01), double-cue (*p* < 0.05), and no-cue conditions (*p* < 0.05).

#### 3.1.2. Accuracy

See Table 2 for the descriptive statistics for accuracy in each condition and its correlation with CDS symptoms. None of the accuracies significantly correlated with CDS symptoms.

#### 3.1.3. Attention Network

None of the attentional network scores correlated with CDS symptoms (see Table 2 for descriptive statistics and the correlation results).

### 3.2. MEG Data

#### 3.2.1. ERF

**Figure 2:**
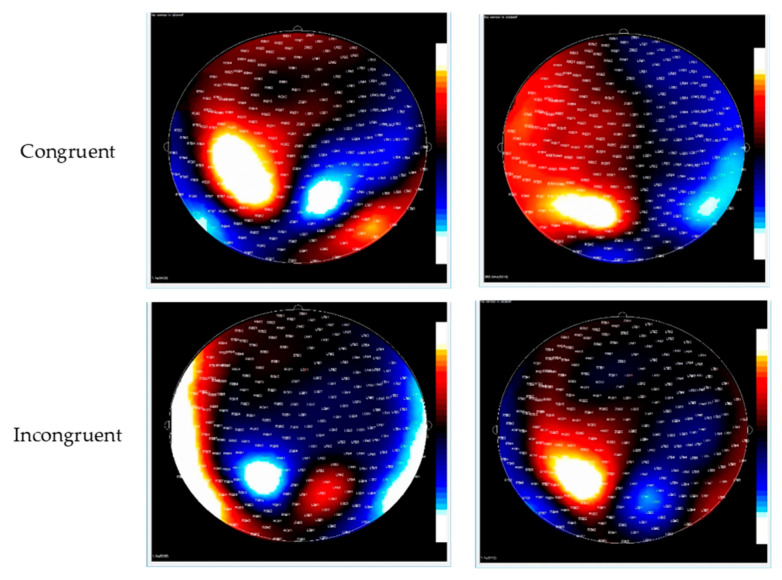
Contour maps of the M2 and M3 component in congruent (**top row**) and incongruent trials (**bottom row**). Red represents the incoming magnetic fields; blue represents outgoing magnetic fields.

**Figure 3:**
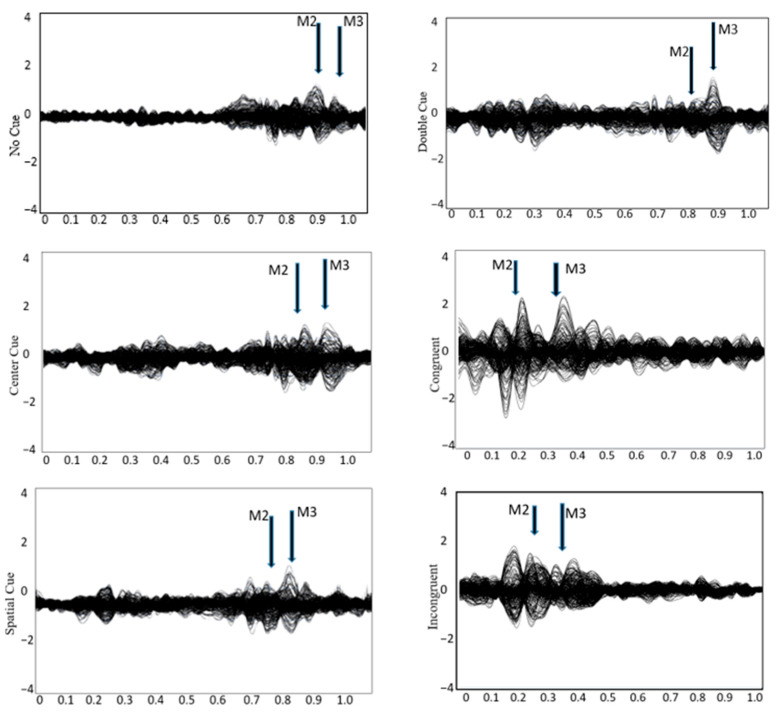
Grandaverage MEG waveform during the attention network test across four cueing conditions: no cue, center cue, spatial cue, and double cue, as well as congruent and incongruent trials. Major neuromagnetic responses, M2 and M3, are identified.

**Figure 4:**
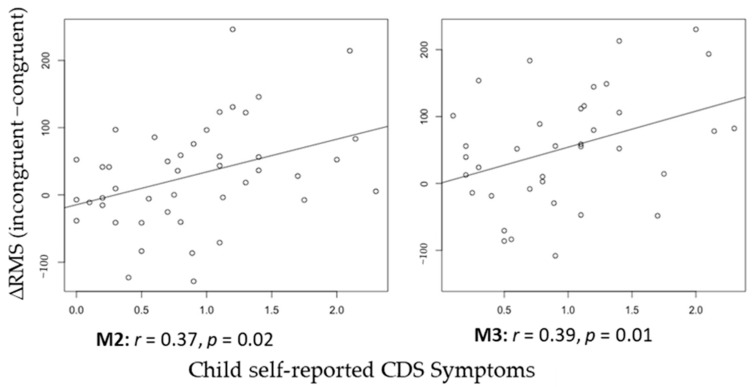
Correlation between child self-reported CDS Symptoms and ∆RMS (incongruent−congruent) of M2 and M3.

See Figure 2 for the topo map for each of the ERFs and Figure 3 for the grand-average MEG waveform. The mean and SD of the RMS of M2 and M3 in different conditions of the ANT are shown in Table 3. Controlling for ADHD status, there was a positive partial correlation between CDS symptoms and ΔRMS (incongruent–congruent) in M2 (i.e., waveform during 150–250 ms after flanker stimulus onset), r(36) = 0.37, *p* = 0.02, and M3 (i.e., waveform during 250–350 ms after flanker stimulus onset), r(36) = 0.39, *p* = 0.01 (Figure 4). That is, even when controlling for ADHD, higher CDS symptom scores were associated with a larger ΔRMS (incongruent–congruent) in M2 and M3. There were no significant partial correlations between self-reported CDS symptoms and ΔRMS (double cue−no cue) in M2, r(36) = 0.05, *p* = 0.91, and M3, r(36) = 0.05, *p* = 0.86, as well as ΔRMS (spatial cue–center cue) in M2, r(36) = 0.08, *p* = 0.62, and M3, r(36) = 0.03, *p* = 0.65.

#### 3.2.2. Source Localization

**Figure 5:**
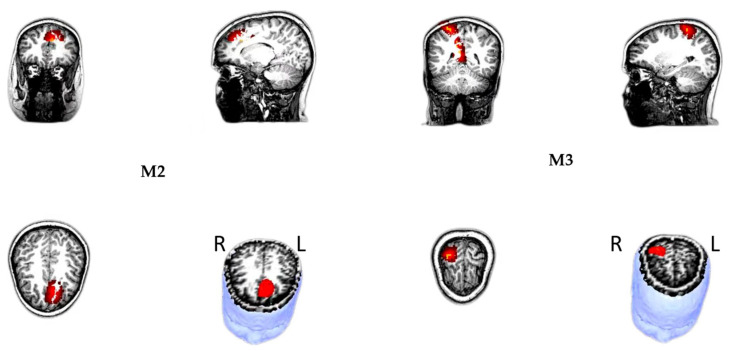
Magnetic source location showed that the M2, M3 originated in the medial prefrontal cortex (mPFC)—**left** and the right superior parietal lobule (SPL)—**right**. All sources were presented on the surface rendition of the corresponding participant’s MR images.

**Figure 6:**
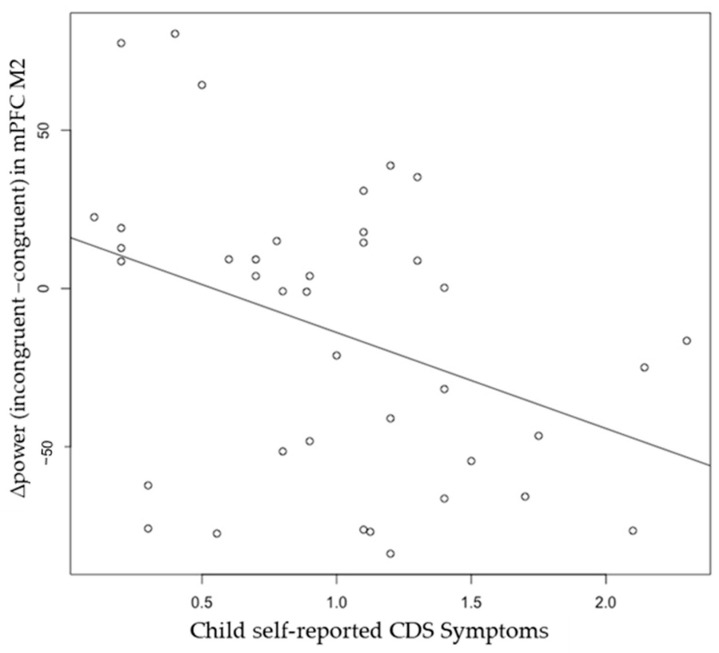
Pearson correlation between child self-reported CDS Symptoms and ∆power (incongruent−congruent) in M2 medial prefrontal cortex, *r*(*37*) = −0.38, *p* = 0.01. The association is no longer significant after controlling for ADHD.

The neuromagnetic sources of M2 and M3 in the congruent and incongruent trials were volumetrically scanned, and constant activation was revealed in the frontal and parietal cortices, especially in the medial prefrontal cortex (mPFC) and right superior parietal lobe (SPL) (Figure 5). The whole-head power was measured with total voxels and averaged voxels. The descriptive statistics for each brain region source are displayed in Table 4. Pearson correlation revealed a significant negative correlation between self-reported CDS symptoms and M2 Δpower (incongruent−congruent) in the mPFC, r(37) = −0.38, *p* = 0.01 (Figure 6), but not in the right SPL. However, this association was no longer significant after controlling for ADHD, r(36) = −0.23, *p* = 0.09. No significant correlations were observed between self-reported CDS symptoms and M3 Δpower (incongruent−congruent) in either the mPFC, r(37) = −0.09, or the right SPL, r(36) = −0.11, *p*s > 0.05.

## 4. Discussion

To our knowledge, the present study is the first neuroimaging study to examine the CDS-related neural correlates of attentional functioning in children. The present study investigated the attentional profile of children performing the ANT using whole-head MEG in a sample recruited to capture the full spectrum of CDS symptoms. The performance in the ANT is largely consistent with that in previous research conducted in children in terms of congruency effect, with faster performance in congruent trails than in incongruent trials, as well as cue effect, with the slowest performance in no-cue trials and the fastest in spatial-cue trials compared with other cuing conditions [48,49,50]. A congruency effect was also observed in accuracy such that the accuracy in congruent trials was higher than that in incongruent trials. Overall, children performed the ANT with a relatively high accuracy. CDS symptom severity was not associated with any behavioral measures of the alerting, orienting, or executive attention networks. However, child self-reported CDS symptom severity was positively correlated with ΔRMS (incongruent–congruent) for M2 and M3. Follow-up source analysis revealed that self-reported CDS symptoms were negatively correlated with the M2 Δpower (incongruent–congruent) in the medial prefrontal cortex (mPFC), suggesting reduced power allocation in a region critical for attentional control. However, this association was no longer significant after controlling for ADHD symptoms, indicating that the observed effect may be accounted for by overlapping variance with ADHD, though this possibility should be considered further in larger samples before drawing firm conclusions.

Our finding showing CDS symptom severity to be associated with a larger ΔRMS (incongruent–congruent) in M2 and M3 suggests that children with CDS may exert more effort in processing incongruent trials. Adults with CDS have been found to show lower efficiency than adults with ADHD in a task examining early and late selective attention processing speed with irrelevant distractors [51]. In line with this prior study [51], our finding suggests that distractors, such as flankers in the incongruent trials of the ANT, may require more mental resources in stimuli processing to maintain the same level of task performance.

In addition, greater CDS symptom severity was associated with reduced power in the mPFC. The mPFC is a region that has consistently been found to be engaged in tasks requiring conflict monitoring [52,53,54]. During the ANT, the mPFC is activated in response to incongruent or conflicting stimuli, where it helps to manage and resolve competing responses by supporting attentional shifts and maintaining focus on task-relevant information. For instance, previous research has identified significant mPFC activation in response to the executive control demands of the ANT, suggesting its role in managing attentional conflicts between competing stimuli [55]. Similarly, the mPFC, particularly the anterior cingulate cortex (part of the mPFC), has also been found to be involved in attentional control and error monitoring, which are essential for adapting responses to challenging or conflicting conditions [56]. Although our initial findings suggested that children with elevated CDS may show reduced mPFC involvement, this association was no longer significant after controlling for ADHD status. This suggests that the observed neural differences may reflect overlapping variance between CDS and ADHD and highlights the need for further research in larger samples to disentangle their distinct neurocognitive profiles.

We did not find the CDS symptoms’ severity to be associated with power in the SPL source localized from M2. The SPL is involved in sensory integration, spatial orientation, and attention shifting, especially in tasks requiring the coordination of visual and spatial information [20]. Although previous fMRI research implicates SPL hypoactivation in CDS during attentional control tasks [15], our MEG source-localized M2-ERF power did not show a significant relationship with CDS symptom severity in the SPL. This suggests that SPL contributions to CDS may depend on specific task demands and later-stage top-down attentional processes not captured in early MEG evoked responses. Future work could explore SPL engagement during tasks that explicitly require spatial attention shifts or sensory integration.

Contrary to the possibility that CDS is related to deficient vigilance and orientation [13], the present study did not find CDS symptom severity to be associated with any of the behavioral measures of the three attentional networks. This aligns with two previous studies conducted in primary-school children from the general population [22,23]. This suggests that CDS symptoms may not directly impact the efficiency of the alerting, orienting, or executive control networks as measured by behavioral performance. Alternatively, the relatively high accuracy in the current ANT suggests that the task might be too easy to engage attentional resources, resulting in behavioral measures that are insufficiently sensitive enough to capture attention network deficits.

Several limitations are important to note. First, the sample primarily comprised non-Hispanic White children from higher-income families. Future research should recruit more diverse and representative samples to improve the generalizability of the findings. Second, while the study included children with a full range of CDS symptoms, a large percentage met the criteria for ADHD, which may have influenced the results. This study did not collect a child self-report measure of ADHD symptoms, and it will be important for future studies to control for ADHD symptoms to isolate the unique neural correlates of CDS. Third, the sample may be considered small in comparison to previous studies examining the neural correlates of CDS [14,57]; thus, studies with larger samples will be needed to replicate and extend our findings. Moreover, it would be beneficial in future studies to have a longer washout period. Lastly, while the current study focused on individual differences using a dimensional approach rather than group-level (case–control) comparisons, the sample is of high heterogeneity. Future research should incorporate a well-matched control group to contextualize these findings and evaluate their specificity to CDS. The frequent co-occurrence of CDS and ADHD (particularly the inattentive type) poses a challenge in isolating the effects uniquely attributable to CDS. Although dimensional symptom measures allowed us to explore associations specific to CDS traits, the presence of comorbid ADHD symptoms may confound interpretations, especially given the shared attentional and executive function impairments observed in both conditions. Future studies with larger samples and stratified designs are needed to better disentangle the unique and overlapping neural correlates of CDS and ADHD.

## 5. Conclusions

This study adds to the remarkably small literature on the neural correlates of CDS. Findings revealed that attention-related deficits in CDS are likely to be associated with altered neural responses during conflict processing, suggesting that children with more severe CDS symptoms may require greater cognitive effort and exhibit reduced engagement of brain regions critical for attentional control. However, the disappearance of mPFC effects after controlling for ADHD symptoms highlights the need to further disentangle the unique and overlapping neural mechanisms of CDS and ADHD, ideally in larger, well-powered samples. These findings are important to inform our understanding of the neural correlates of CDS and to build a neuroscience-based understanding of the attentional profile in CDS.

## Figures and Tables

**Table 1:** Sample characteristics (*n* = 43).

Age (at Inclusion Visit)	
8–12 years (M = 9.95, SD = 1.45)	
**IQ**	
84–135 (M = 109.51, SD = 12.76)	
**Sex**	
Female	*n* = 17 (37.2%)
Male	*n* = 26 (62.8%)
**Race/Ethnicity**	
Black	*n* = 2 (4.7%)
Hispanic	*n* = 1 (2.3%)
Multiracial	*n* = 2 (4.7%)
White	*n* = 38 (88.4%)
**Family Income**	
Up to USD 40,000	*n* = 3 (7.0%)
USD 40,001–USD 80,000	*n* = 10 (23.3%)
USD 80,001–USD 120,000	*n* = 15 (34.9%)
Over USD 120,000	*n* = 15 (34.9%)
**ADHD Presentation**	
Inattentive presentation	*n* = 20 (46.5%)
Combined presentation	*n* = 6 (14.0%)
No ADHD	*n* = 17 (39.5%)
**Other Diagnoses**	
Oppositional defiant disorder	*n* = 4 (9.3%)
Generalized anxiety disorder	*n* = 2 (4.7%)
Social anxiety disorder	*n* = 2 (4.7%)
Specific phobia	*n* = 1 (4.7%)
Dysthymia	*n* = 1 (2.3%)

Note. All of the other diagnoses were present in the ADHD subgroup (*n* = 26). ADHD = attention-deficit/hyperactivity disorder; IQ = intelligence quotient.

**Table 2:** Attention network score, mean of the median RT (milliseconds) and accuracy per task. Condition of the attention network test and their correlations with cognitive disengagement syndrome (CDS).

Congruency	Cue	RT (ms)		Accuracy (100%)		Partial Correlation Between RT and CDS		Partial Correlation Between Accuracy and CDS	
		*M*	*SD*	*M*	*SD*	*r*	*p*	*r*	*p*
Congruent	No cue	666.16	81.97	0.89	0.10	−0.11	0.48	0.03	0.84
	Center cue	645.81	93.44	0.90	0.10	0.01	0.91	0.02	0.87
	Double cue	637.92	88.05	0.90	0.10	0.05	0.97	−0.03	0.83
	Spatial cue	605.07	80.36	0.92	0.08	−0.001	0.99	−0.06	0.69
Incongruent	No cue	717.11	85.60	0.86	0.12	−0.04	0.82	−0.02	0.89
	Center cue	702.05	84.62	0.86	0.13	−0.02	0.91	−0.03	0.87
	Double cue	689.89	92.94	0.90	0.10	−0.09	0.56	−0.04	0.83
	Spatial cue	634.87	87.81	0.92	0.09	−0.04	0.79	0.02	0.88
Attention network score	Alerting	27.73	30.03	–	–	−0.07	0.65	–	–
	Orienting	53.96	30.96	–	–	0.07	0.68	–	–
	Executive	47.24	25.28	–	–	−0.10	0.54	–	–

**Table 3:** Mean and SD of the RMS for M2 and M3 across conditions in the attention network test.

Condition	M2		M3	
	*M*	*SD*	*M*	*SD*
No cue	194.01	57.18	223.30	74.50
Center cue	210.30	64.10	241.46	82.17
Double cue	229.37	85.13	226.00	58.31
Spatial cue	214.41	67.23	214.74	69.61
Congruent	182.34	94.43	184.71	88.72
Incongruent	212.98	79.50	239.98	79.50

**Table 4:** Power of M2 and M3 in the frontal and parietal region per congruency.

ERF	Region of Interest	Congruency	*M*	*SD*
M2	Medial prefrontal cortex	Congruent	60.58	17.90
		Incongruent	45.33	34.62
	Right superior parietal lobe	Congruent	72.28	22.60
		Incongruent	73.56	18.64
M3	Medial prefrontal cortex	Congruent	45.95	27.98
		Incongruent	39.71	34.50
	Right superior parietal lobe	Congruent	74.21	21.94
		Incongruent	69.79	21.72

## Data Availability

Data is available from the last author upon reasonable request and the establishment of a data sharing agreement.

## Abbreviations

The following abbreviations are used in this manuscript:

CDS	Cognitive Disengagement Syndrome
SCT	Sluggish Cognitive Tempo
ADHD	Attention-Deficit/Hyperactivity Disorder
ANT	Attention Network Test
